# Sex-stratified analysis of marketing exposure and current e-cigarette use among Saudi adolescents

**DOI:** 10.3389/fpubh.2025.1649537

**Published:** 2025-08-04

**Authors:** Najim Z. Alshahrani, Abdullah M. Alarifi, Mohammed R. Algethami, Mohammed A. Aljunaid, Ahmed K. Shukri, Mohannad A. Alzain

**Affiliations:** ^1^Department of Family and Community Medicine, Faculty of Medicine, University of Jeddah, Jeddah, Saudi Arabia; ^2^Deputyship of Population Health, Ministry of Health, Riyadh, Saudi Arabia; ^3^Department of Family Medicine, Faculty of Medicine, King Abdulaziz University, Jeddah, Saudi Arabia

**Keywords:** e-cigarettes, adolescents, tobacco marketing, Saudi Arabia, Global Youth Tobacco Survey, nicotine use, POS advertisement, youth smoking prevention

## Abstract

**Background:**

The rising use of e-cigarettes among adolescents presents a growing public health concern, particularly in countries like Saudi Arabia, where tobacco marketing regulation is still evolving. Although marketing is a known driver of youth tobacco uptake, evidence from the Eastern Mediterranean region remains limited. This study examined the association between exposure to e-cigarette marketing and current use among Saudi adolescents using nationally representative data.

**Methods:**

We analysed cross-sectional data from the 2022 Global Youth Tobacco Survey (GYTS) in Saudi Arabia, a nationally representative, school-based survey of adolescents aged 13–15 years. Key exposures included seeing a point-of-sale (POS) advertisement and being offered a free e-cigarette. A composite marketing exposure score (0, 1, or 2 exposures) was created. The outcome was current e-cigarette use, defined as use on at least one day in the past 30 days. Survey-weighted logistic regression models estimated crude and adjusted odds ratios (aORs), adjusting for age, sex, parental smoking, and peer smoking. Sex-stratified analyses were also conducted.

**Results:**

Among 5,610 adolescents, 300 (5.4%) reported current e-cigarette use. Of all respondents, 5.7% had been offered a free e-cigarette and 19.7% had seen a POS advertisement. Both exposures were significantly associated with current e-cigarette use: free product offer (aOR: 6.57; 95% CI: 4.61–9.36; *p* < 0.001) and POS ad exposure (aOR: 2.66; 95% CI: 1.79–3.97; *p* < 0.001). A dose–response relationship was observed, with those exposed to both forms of marketing having 15 times the odds of current use (aOR: 15.05; 95% CI: 7.81–29.02; *p* < 0.001). Associations were significant for both males and females.

**Conclusion:**

Exposure to e-cigarette marketing is a strong and consistent predictor of adolescent use in Saudi Arabia. These findings support urgent policy action to restrict youth-targeted tobacco promotions.

## Introduction

Tobacco use among adolescents remains a critical public health concern worldwide, with the proliferation of alternative nicotine products such as electronic cigarettes (e-cigarettes) adding new complexities to tobacco control efforts (1–3). E-cigarettes are often marketed as harm-reduction tools for adult smokers, yet their growing popularity among youth, many of whom have never smoked conventional cigarettes, raises questions about their role in promoting nicotine addiction in new generations (3). Globally, increasing numbers of adolescents are experimenting with or regularly using e-cigarettes, often influenced by product appeal, social trends, and accessibility (1, 2). Evidence suggests that adolescents are especially susceptible to marketing tactics due to the developmental vulnerability of their decision-making processes (4–7), and the normalisation of vaping in youth culture has heightened concerns about the long-term public health implications.

E-cigarettes were first introduced in the early 2000s as an alternative to combustible cigarettes and have since evolved into a wide range of devices, including disposable e-cigarettes, rechargeable pod systems, and tank-style vaporizers (3). In Saudi Arabia, the e-cigarette market has expanded considerably over the past decade. One of the most influential drivers of e-cigarette uptake among adolescents is exposure to tobacco marketing, which includes direct promotions (such as being offered free samples) and indirect promotions (such as advertisements at the point of sale or on social media) (8–10). Unlike traditional cigarette marketing, which is heavily restricted in many countries, e-cigarette promotions often exploit regulatory loopholes, particularly in low- and middle-income settings (11–14). Studies in high-income countries, including the United States and the United Kingdom, have consistently shown that exposure to tobacco marketing significantly increases the odds of e-cigarette use among youth (8, 15). However, there remains a paucity of evidence from the Eastern Mediterranean region, including Saudi Arabia, where cultural, regulatory, and commercial environments differ significantly from those in Western contexts. Understanding the impact of marketing in this region is crucial for tailoring tobacco control policies and protecting adolescents from nicotine initiation.

Saudi Arabia has experienced a rapid transformation in tobacco product availability and marketing in recent years, including the legalisation and commercial distribution of e-cigarettes (16). At the same time, the country remains committed to the World Health Organization Framework Convention on Tobacco Control (WHO FCTC), which urges parties to restrict all forms of tobacco advertising, promotion, and sponsorship (17). Despite this, adolescents in Saudi Arabia may still be exposed to e-cigarette marketing in retail environments, through peers, or via informal networks. Given the rising prevalence of e-cigarette use among Saudi youth and the documented role of marketing in shaping health behaviours, it is essential to generate empirical evidence to inform national prevention strategies. This is especially relevant in a setting where youth tobacco use is influenced not only by marketing but also by social and familial contexts.

The present study aimed to examine the association between exposure to e-cigarette marketing and current e-cigarette use among adolescents aged 13–15 years in Saudi Arabia, using nationally representative data from the 2022 Global Youth Tobacco Survey (GYTS). Specifically, we assessed the independent associations of two forms of marketing exposure, being offered a free e-cigarette and seeing a point-of-sale advertisement, with e-cigarette use, including stratified analyses by sex. We also explored the dose–response relationship between cumulative marketing exposure and current use. This evidence is intended to guide policymakers and public health professionals in designing effective, context-specific interventions to limit youth exposure to tobacco marketing in Saudi Arabia.

## Methods

### Study design and population

This cross-sectional study was based on data from the 2022 Global Youth Tobacco Survey (GYTS) conducted in Saudi Arabia (18). The GYTS is a nationally representative, school-based survey developed by the World Health Organization and the US Centers for Disease Control and Prevention (18). It targets adolescents aged 13–15 years to monitor tobacco use behaviours, exposure to pro- and anti-tobacco influences, and key social determinants. The survey employed a two-stage cluster sampling design. In the first stage, schools were selected with a probability proportional to enrolment size. In the second stage, classes were randomly selected within each school, and all students in the selected classes were eligible to participate. The questionnaire was self-administered, anonymous, and completed during a regular classroom period. The total sample consisted of 5,610 students aged 13–15 years.

### Exposure

The primary exposures of interest were indicators of tobacco marketing exposure. Exposure to point-of-sale (POS) advertisements was measured using the question: “During the past 30 days, did you see any advertisements or promotions for tobacco products at points of sale (such as grocery stores, shops, kiosks, etc.)?” Responses were categorised as “Saw POS ad” (Yes), “No ad seen” (No), or “Did not visit any points of sale,” with the latter treated as missing. The second exposure focused on whether respondents had been offered a free electronic cigarette or vaping device by a tobacco company representative in the past 30 days, as captured by variable CR35. This was coded as a binary variable (Yes or No). To assess cumulative exposure, a composite marketing exposure score was constructed by summing the number of exposure types reported: a score of 0 indicated no exposure, 1 indicated exposure to one type (either POS advertisement or free product offer), and 2 indicated exposure to both. This score was used to evaluate a potential dose–response relationship between marketing exposure and current e-cigarette use.

### Outcome

The main outcome variable was current e-cigarette use. This was derived from a survey question asking respondents on how many days in the past 30 days they had used an e-cigarette or vaping product. For analysis, responses were dichotomised into “current use” (defined as use on one or more days in the past 30 days) and “non-use” (defined as no use in the past 30 days). This operational definition is consistent with international surveillance standards for adolescent tobacco use and allows for the identification of recent users likely influenced by recent exposures (19).

### Covariates

Several covariates were included in the regression models to account for potential confounding. Age was categorised as 13, 14, or 15 years and included as a categorical variable. Sex was coded as male or female based on self-report. Parental smoking status was assessed by asking whether either parent smoked tobacco; responses were dichotomised into “no parent smokes” and “at least one parent smokes.” Peer smoking was measured by asking whether any of the respondent’s closest friends smoked cigarettes or other tobacco products. Responses were categorised similarly as “no friends smoke” and “at least one friend smokes.” These covariates were selected *a priori* based on prior evidence linking them to youth tobacco use and marketing susceptibility (6, 20).

Missing data in the covariates were handled using listwise deletion. The overall proportion of missing data was low (<5% across all key variables), and we assumed that data were missing at random. Given the small extent of missingness, the likelihood of bias introduced by listwise deletion is minimal.

### Statistical analyses

All statistical analyses accounted for the complex sampling design of the GYTS, including the use of sampling weights, stratification, and clustering at the primary sampling unit (PSU) level. Descriptive statistics were used to summarise the sample characteristics by computing unweighted frequencies and weighted percentages. To examine associations between tobacco marketing exposures and current e-cigarette use, survey-weighted logistic regression models were estimated. Crude odds ratios (ORs) with 95% confidence intervals were derived from univariable models for each exposure. Adjusted odds ratios (aORs) were obtained from multivariable models controlling for age, sex, parental smoking, and peer smoking. To explore whether associations differed by sex, stratified analyses were performed for males and females separately. For the marketing exposure score, additional models assessed potential dose–response effects by comparing adolescents with 0, 1, or 2 types of marketing exposure. The reference group in these models was those with no exposure. All analyses used two-sided statistical tests, with significance set at *p* < 0.05. Listwise deletion was applied in all regression models, and multicollinearity between covariates was assessed using Variance Inflation Factors (VIF), with all VIFs < 5. All analyses were conducted using Stata version 17.0, and results were presented in accordance with STROBE guidelines for reporting observational studies.

## Results

Table 1 presents the descriptive characteristics of the unweighted sample of 5,610 adolescents aged 13–15 years who participated in the 2022 Global Youth Tobacco Survey (GYTS) in Saudi Arabia. The largest age group was 14-year-olds, comprising 37.0% of the weighted sample, followed by 13-year-olds (34.4%) and 15-year-olds (28.6%). The sex distribution was relatively balanced, with males accounting for 50.7% and females 49.3% of respondents. Most adolescents reported that no parent smoked (83.0%), while 17.0% indicated that at least one parent used tobacco. In contrast, 16.0% reported having one or more close friends who smoked, suggesting lower but still notable peer influence. Regarding exposure to tobacco marketing, 19.7% of adolescents had seen a point-of-sale (POS) advertisement for e-cigarettes in the past 30 days. A smaller proportion (5.7%) reported being offered a free e-cigarette by a tobacco company representative. When combining these indicators into a marketing exposure score, 78.5% of respondents reported no exposure to either marketing type, 19.9% were exposed to one type, and only 1.7% had encountered both. Only 5.4% are current e-cigarette users.

**Table 1:** Characteristics of adolescents aged 13–15 in Saudi Arabia (GYTS 2022).

Variable	Category	Unweighted n	Weighted %
Age			
	13 years old	1,928	34.4%
	14 years old	2,140	37.0%
	15 years old	1,542	28.6%
Sex			
	Male	2,900	50.7%
	Female	2,658	49.3%
Parental smoking			
	No parent smokes	4,482	83.0%
	≥1 parent smokes	915	17.0%
Peer smoking			
	No friends smoke	4,694	84.0%
	≥1 friend smokes	887	16.0%
Saw point of Sale advert			
	No ad seen	3,096	80.3%
	Saw POS ad	768	19.7%
Offered free e-cigarette			
	Not offered	5,151	94.3%
	Offered	314	5.7%
Marketing exposure score			
	0 exposures	2,956	78.5%
	1 exposure	757	19.9%
	2 exposures	63	1.7%
Current e-cigarette use			
	No	5,217	94.6
	Yes	300	5.4

Missing data—Sex: *n* = 52; Parental smoking: *n* = 213; Peer smoking: *n* = 29; POS ad exposure: *n* = 1,746; Offered e-cigarette: *n* = 145; Marketing exposure score: *n* = 1,834.

Table 2 presents the associations between tobacco marketing exposure and current e-cigarette use among adolescents aged 13–15 years in Saudi Arabia. In univariable survey-weighted logistic regression models, adolescents who had been offered a free e-cigarette were over seven times more likely to report current use of e-cigarettes compared to those who had not (crude OR = 7.31; 95% CI: 5.32–10.05; *p* < 0.001). Similarly, exposure to a point-of-sale (POS) advertisement for e-cigarettes was associated with a nearly four-fold increase in the odds of current use (crude OR = 3.73; 95% CI: 2.48–5.60; *p* < 0.001). These associations remained statistically significant after adjusting for potential confounders including age, sex, parental smoking, and peer smoking. The adjusted odds ratio for receiving a free e-cigarette offer was 6.57 (95% CI: 4.61–9.36; *p* < 0.001), while exposure to POS advertisements was associated with an adjusted OR of 2.66 (95% CI: 1.79–3.97; *p* < 0.001).

**Table 2:** Association between tobacco marketing exposure and current e-cigarette use among adolescents aged 13–15 in Saudi Arabia (GYTS 2022).

Predictor variable	Crude OR (95% CI)	*p*-value	Adjusted OR (95% CI)	*p*-value
Offered free e-cigarette	7.31 (5.32–10.05)	<0.001	6.57 (4.61–9.36)	<0.001
Saw Point of Sale advertisement	3.73 (2.48–5.60)	<0.001	2.66 (1.79–3.97)	<0.001

Crude odds ratios (ORs) were estimated using univariable survey-weighted logistic regression. Adjusted ORs were estimated using multivariable models controlling for age, sex, parental smoking, and peer smoking.

Table 3 displays the sex-stratified associations between tobacco marketing exposure and current e-cigarette use among adolescents. Among male adolescents, being offered a free e-cigarette was strongly associated with current use, with an adjusted odds ratio (aOR) of 5.60 (95% CI: 3.71–8.47; *p* < 0.001). Exposure to a point-of-sale (POS) advertisement was also significantly associated with current e-cigarette use among males (aOR = 3.12; 95% CI: 1.82–5.34; *p* < 0.001). Among female adolescents, the strength of the association between being offered a free e-cigarette and current use was even more pronounced, with an aOR of 8.53 (95% CI: 4.41–16.47; *p* < 0.001). POS advertisement exposure was also significantly associated with e-cigarette use among females, though the magnitude was slightly lower than in males (aOR = 2.13; 95% CI: 1.15–3.97; *p* = 0.018). These sex-specific models were adjusted for age, parental smoking, and peer smoking, and all estimates account for the complex survey design.

**Table 3:** Association between tobacco marketing exposure and current e-cigarette use, stratified by sex (GYTS 2022).

Predictor variable	aOR (95% CI)—Males	*p*-value	aOR (95% CI)—Females	*p*-value
Offered free e-cigarette	5.60 (3.71–8.47)	<0.001	8.53 (4.41–16.47)	<0.001
Saw POS advertisement	3.12 (1.82–5.34)	<0.001	2.13 (1.15–3.97)	0.018

Estimates are based on survey-weighted logistic regression models adjusted for age, parental smoking, and peer smoking.

Table 4 presents the association between cumulative marketing exposure and current e-cigarette use among adolescents aged 13–15. Compared to those with no exposure to tobacco marketing, adolescents with one type of exposure (either being offered a free e-cigarette or seeing a point-of-sale advertisement) had significantly higher odds of current e-cigarette use. The crude odds ratio (OR) was 3.80 (95% CI: 2.57–5.61; *p* < 0.001), and this association remained significant after adjustment for age, sex, parental smoking, and peer smoking (adjusted OR = 2.65; 95% CI: 1.81–3.88; *p* < 0.001). Adolescents exposed to both forms of marketing exhibited substantially elevated odds of e-cigarette use. The crude OR for those with two exposure types was 20.21 (95% CI: 10.92–37.40; *p* < 0.001), which decreased but remained extremely strong in the adjusted model (aOR = 15.05; 95% CI: 7.81–29.02; *p* < 0.001). These findings underline a clear dose–response relationship between cumulative marketing exposure and e-cigarette use, highlighting the additive risk posed by multiple forms of promotional contact.

**Table 4:** Association between e-cigarette marketing exposure and current e-cigarette use among adolescents aged 13–15 in Saudi Arabia (GYTS 2022).

Marketing exposure score	Crude OR (95% CI)	*p*-value	Adjusted OR (95% CI)	*p*-value
1 exposure type	3.80 (2.57–5.61)	<0.001	2.65 (1.81–3.88)	<0.001
2 exposure types	20.21 (10.92–37.40)	<0.001	15.05 (7.81–29.02)	<0.001

Estimates are from survey-weighted logistic regression models (reference: 0 exposure). Adjusted models control for age, sex, parental smoking, and peer smoking. Analyses account for complex survey design.

Table 5 shows the sex-stratified associations between tobacco marketing exposure and current e-cigarette use among adolescents. Among males, exposure to one type of tobacco marketing was associated with a significantly increased likelihood of current e-cigarette use, with an adjusted odds ratio (aOR) of 3.09 (95% CI: 1.82–5.24; *p* < 0.001). The risk was substantially higher for those exposed to both marketing types, with an aOR of 21.13 (95% CI: 7.79–57.31; *p* < 0.001). A similar pattern was observed among females, although the magnitudes of association were comparatively lower. Female adolescents who reported one type of exposure had a significantly higher odds of current e-cigarette use (aOR = 2.10; 95% CI: 1.14–3.89; *p* = 0.019), while those with both exposures showed a markedly elevated association (aOR = 10.76; 95% CI: 4.33–26.75; *p* < 0.001).

**Table 5:** Association between marketing exposure and current e-cigarette use, stratified by sex (GYTS 2022).

Marketing exposure	Males (aOR, 95% CI)	*p*-value	Females (aOR, 95% CI)	*p*-value
1 exposure type	3.09 (1.82–5.24)	<0.001	2.10 (1.14–3.89)	0.019
Both exposure types	21.13 (7.79–57.31)	<0.001	10.76 (4.33–26.75)	<0.001

Estimates adjusted for age, parental smoking, and peer smoking. Models are stratified by sex and account for the complex survey design using appropriate weights, strata, and PSUs.

## Discussion

This study provides nationally representative evidence on the association between tobacco marketing exposure and current e-cigarette use among adolescents in Saudi Arabia. The results indicate that exposure to two forms of e-cigarette marketing, being offered a free sample and seeing a point-of-sale advertisement, are each strongly associated with current e-cigarette use among adolescents aged 13–15 years. These findings align with existing research from other countries, where marketing exposure has consistently been identified as a powerful driver of youth vaping (15, 21–23). However, this is one of the first studies to demonstrate these associations in the Middle East, where marketing regulations are evolving, and cultural norms around tobacco use differ from those in Western settings. The magnitude of the associations observed suggests that marketing influences adolescent behaviour in Saudi Arabia in ways that are comparable to or stronger than in other regions, despite differing regulatory environments.

The observed dose–response relationship between cumulative marketing exposure and current e-cigarette use adds further weight to concerns about youth susceptibility to (tobacco) industry tactics. Adolescents who reported both seeing a retail advertisement and being offered a free e-cigarette were more than 15 times as likely to use e-cigarettes compared to those with no marketing exposure. This pattern suggests that marketing exposures may not operate in isolation but rather have a compounding effect when experienced simultaneously. These findings are consistent with the “cumulative risk” model in public health, which proposes that the accumulation of multiple environmental or social risks can greatly increase the likelihood of harmful behaviours (24, 25). In the case of Saudi adolescents, limited restrictions on retail advertising or free product offers may create an enabling environment for nicotine experimentation, especially in the absence of strong counter-marketing campaigns.

Sex-stratified analyses revealed that both males and females were influenced by tobacco marketing, but there were important nuances. Among females, being offered a free e-cigarette was associated with a higher adjusted odds of use compared to males, suggesting that direct promotional tactics may be particularly persuasive among adolescent girls. While males demonstrated stronger associations in the dose–response analysis, the differences may reflect variations in social norms, access to marketing channels, or responsiveness to peer influence across sexes. These findings are relevant in the Saudi context, where traditional gender roles and tobacco norms have historically differed, but where recent years have seen rapid changes in youth culture and media exposure. Targeted interventions must therefore consider these gendered dynamics when addressing e-cigarette uptake.

Another important contextual factor is the rising popularity of e-cigarettes among youth in many countries, often framed around lifestyle appeal, flavour diversity, and the perception that vaping is safer than traditional smoking (26–28). In Saudi Arabia, where smoking remains a leading contributor to non-communicable diseases (29), there is an urgent need to prevent nicotine uptake in new generations. Although the country is a signatory to the WHO Framework Convention on Tobacco Control, regulatory enforcement and awareness regarding novel tobacco products may be inconsistent. These findings highlight an important policy gap, particularly concerning the marketing of e-cigarettes in retail settings and through direct product distribution to minors. Without stricter controls and enforcement mechanisms, marketing-driven uptake of e-cigarettes among adolescents may continue to rise (30, 31).

### Public health implications

The results of this study have significant implications for tobacco control policy in Saudi Arabia and comparable settings. Limiting adolescent exposure to both direct and indirect forms of e-cigarette marketing should be prioritised. Public health authorities should strengthen the implementation and enforcement of existing advertising bans to include e-cigarettes and prohibit promotional giveaways targeted at minors. School-based prevention programmes should be expanded to include content that explicitly addresses the influence of marketing and teaches media literacy skills to counter industry messaging. Furthermore, comprehensive surveillance systems must monitor not only prevalence but also the evolving landscape of marketing channels influencing youth.

### Strengths and limitations

This study has several strengths, including its use of a large, nationally representative sample and its application of rigorous survey-weighted analyses that account for the complex sampling design of the GYTS. The inclusion of both individual marketing exposures and a cumulative score allowed for a robust exploration of dose–response relationships. Stratified analyses by sex further enriched the understanding of how marketing may differentially affect boys and girls. However, some limitations must be acknowledged. The cross-sectional nature of the data prevents causal inference, and the possibility of reverse causation cannot be ruled out (32–34). Additionally, self-reported data may be subject to recall or social desirability bias, particularly in a cultural context where tobacco use may be stigmatised. Finally, the study did not capture online marketing exposures, which are increasingly common and influential among adolescents.

## Conclusion

This study provides robust evidence that exposure to e-cigarette marketing is strongly associated with current e-cigarette use among adolescents aged 13–15 in Saudi Arabia. Both direct marketing, such as being offered a free e-cigarette, and indirect marketing, such as point-of-sale advertisements, were independently and jointly associated with increased odds of current use. The presence of a dose–response relationship further reinforces concerns about the cumulative impact of multiple marketing exposures on youth behaviour. These findings remain consistent across sex-stratified analyses, though the strength of associations varied between boys and girls, underscoring the need for gender-sensitive prevention strategies. In a context where the commercial availability of e-cigarettes is expanding and regulatory enforcement remains limited, this study highlights a critical gap in adolescent tobacco control. Comprehensive policy action is needed to restrict all forms of tobacco product marketing, particularly those targeting or reaching young people. In addition, school-based and community-level interventions should be strengthened to raise awareness about the risks of nicotine use and to build resilience against industry influence. As Saudi Arabia continues to align its tobacco control efforts with international frameworks such as the WHO Framework Convention on Tobacco Control, it is essential to prioritise youth protection from emerging forms of tobacco promotion. These findings offer timely and context-specific evidence to guide such action, with the goal of preventing early nicotine initiation and promoting long-term public health.

## Data Availability

Publicly available datasets were analysed in this study. This data can be found here: the data can be accessed at the WHO Global Youth Tobacco Survey repository: https://extranet.who.int/ncdsmicrodata/index.php/catalog/966/data-dictionary/F1?file_name=SAU2022_GYTS.
